# Recombinant Antigens from *Phlebotomus perniciosus* Saliva as Markers of Canine Exposure to Visceral Leishmaniases Vector

**DOI:** 10.1371/journal.pntd.0002597

**Published:** 2014-01-02

**Authors:** Jan Drahota, Ines Martin-Martin, Petra Sumova, Iva Rohousova, Maribel Jimenez, Ricardo Molina, Petr Volf

**Affiliations:** 1 Department of Parasitology, Faculty of Science, Charles University in Prague, Prague, Czech Republic; 2 Unidad de Entomología Médica, Servicio de Parasitología, Centro Nacional de Microbiología, Instituto de Salud Carlos III, Majadahonda, Madrid, Spain; National Institutes of Health, United States of America

## Abstract

**Background:**

*Phlebotomus perniciosus* is the main vector in the western Mediterranean area of the protozoan parasite *Leishmania infantum*, the causative agent of canine and human visceral leishmaniases. Infected dogs serve as a reservoir of the disease, and therefore measuring the exposure of dogs to sand fly bites is important for estimating the risk of *L. infantum* transmission. In bitten hosts, sand fly saliva elicits a specific antibody response that reflects the intensity of sand fly exposure. As screening of specific anti-saliva antibodies is limited by the availability of salivary gland homogenates, utilization of recombinant salivary proteins is a promising alternative. In this manuscript we show for the first time the use of recombinant salivary proteins as a functional tool for detecting *P. perniciosus* bites in dogs.

**Methodology/Principal Findings:**

The reactivity of six bacterially-expressed recombinant salivary proteins of *P. perniciosus*, yellow-related protein rSP03B, apyrases rSP01B and rSP01, antigen 5-related rSP07, ParSP25-like protein rSP08 and D7-related protein rSP04, were tested with sera of mice and dogs experimentally bitten by this sand fly using immunoblots and ELISA. In the immunoblots, both mice and canine sera gave positive reactions with yellow-related protein, both apyrases and ParSP25-like protein. A similar reaction for recombinant salivary proteins was observed by ELISA, with the reactivity of yellow-related protein and apyrases significantly correlated with the antibody response of mice and dogs against the whole salivary gland homogenate.

**Conclusions/Significance:**

Three recombinant salivary antigens of *P. perniciosus*, yellow-related protein rSP03B and the apyrases rSP01B and rSP01, were identified as the best candidates for evaluating the exposure of mice and dogs to *P. perniciosus* bites. Utilization of these proteins, or their combination, would be beneficial for screening canine sera in endemic areas of visceral leishmaniases for vector exposure and for estimating the risk of *L. infantum* transmission in dogs.

## Author Summary

The protozoan parasite *Leishmania infantum* is a causative agent of zoonotic visceral leishmaniasis, an important and potentially fatal human disease. The main reservoir hosts of this *Leishmania* species are dogs, and the only proven vectors are phlebotominae sand flies, *Phlebotomus perniciosus* being considered the major vector in the western Mediterranean area. During feeding on the host, sand flies spit saliva into the host skin; hosts develop a specific antibody response directed against sand fly salivary proteins and levels of these antibodies reflect the intensity of sand fly exposure. As the availability of salivary gland homogenate is limited, recombinant salivary proteins have been suggested as antigens suitable for measuring specific antibody levels. In the present work, we expressed six of the most-antigenic salivary proteins, and studied the mice and canine humoral immune responses to these recombinant proteins. We demonstrated that three proteins, a yellow-related protein and two apyrases, are suitable antigens for measuring anti-*P. perniciosus* antibody levels and estimating the host exposure to this sand fly species.

## Introduction

Canine leishmaniasis (CanL) is a systemic disease with variable clinical symptoms. Its causative agent, the protozoan parasite *Leishmania infantum*, is transmitted by phlebotomine sand flies (Diptera: *Phlebotominae*). CanL occurs frequently around the Mediterranean Basin and in many countries in Latin America, where the prevalence of infection often exceeds 25%. Dogs with inapparent infections often play a role in the circulation of the parasite, as they are able to infect sand flies (reviewed in [1]). New cases of autochthonous leishmaniasis caused by *L. infantum* have been occurring in various countries, suggesting an expansion of CanL towards new biotopes at higher latitudes and higher altitudes (reviewed in [2], [3], [4]). Importantly, CanL is not just a veterinary problem; infected dogs serve as a reservoir host of human visceral leishmaniasis and there is a correlation between the prevalence of leishmaniasis in the canine population and the human disease in many countries [1].

Two sand fly genera are involved in *L. infantum* transmission, *Lutzomyia* in the New World and *Phlebotomus* in the Old World. Seven species of the genus *Phlebotomus*, subgenus *Larroussius*, are proven or probable vectors of CanL in different places around the Mediterranean Basin [5]. Of these, *Phlebotomus perniciosus* has the widest distribution, with ranges in both the southern and northern parts of the Mediterranean, from Morocco and Portugal in the west to Italy in the east and Germany in the north [5].

Measuring the exposure of dogs to sand fly bites is important for estimating the risk of *L. infantum* transmission. Recently, it has been demonstrated that experimental exposure of dogs to *L. longipalpis* or *P. perniciosus* bites elicits the production of specific anti-saliva IgG (measured by ELISA with whole SGH ) that positively correlates with the number of sand fly bites [6], [7]. The elicitation of IgG antibody levels persists for at least five weeks after last exposure of dogs to *P. perniciosus* [7] or even nineteen weeks after dogs are exposed to *L. longipalpis* [6]. Therefore, monitoring canine IgG levels specific for sand fly saliva could indicate their exposure to sand fly bites. Such a monitoring technique would be useful for evaluating the need for, and effectiveness of, anti-vector campaigns [6], [8]. However, obtaining sufficient native antigens through sand fly dissections for the upscaled production of such antigens necessary for ELISA is not feasible; thus there is a need to replace native antigens in the ELISAs by recombinant antigens. To date, recombinant salivary proteins from *Phlebotomus papatasi* have been used to detect the antibody response in mice experimentally exposed to this sand fly species [9], and *L. longipalpis* recombinant antigens have been tested for reactivity with the sera of naturally bitten humans, dogs and foxes [10], [11].

Recent studies [7], [12] have shown that the sera of dogs bitten by *P. perniciosus* recognize up to thirteen antigens in the salivary gland homogenate (SGH) of this species. The most intense reaction has repeatedly been observed against the 43 kDa yellow-related protein PpeSP03B (referred to further in the text as yellow protein rSP03B), the 35.3 kDa PpeSP01B and 35.5 kDa PpeSP01 kDa apyrases (apyrase rSP01B and rSP01, respectively), the 30 kDa antigen 5-related protein PpeSP07 (antigen 5 rSP07), the 29 kDa ParSP25-like protein PpeSP08 (ParSP25 protein rSP08), and the 24.5 kDa D7-related salivary protein PpeSP04 (D7 protein rSP04). The recombinant forms of these six proteins were therefore chosen in the present work as the most promising candidates for markers of *P. perniciosus* exposure.

## Materials and Methods

### 1. Ethics statement

SKH1-hr mice were maintained and handled in the animal facility of Charles University in Prague in accordance with institutional guidelines and Czech legislation (Act No. 246/1992 coll. on Protection of Animals against Cruelty in present statutes at large), which complies with all relevant European Union and international guidelines for experimental animals. The experiments were approved by the Committee on the Ethics of Animal Experiments of the Charles University in Prague (Permit Number: 24773/2008-10001) and were performed under the Certificate of Competency (Registration Number: CZU 934/05; CZU 307/09) in accordance with the Examination Order approved by Central Commission for Animal Welfare of the Czech Republic.

### 2. Sand flies and salivary gland homogenate

Two *Phlebotomus* (*Larroussius*) *perniciosus* sand fly colonies originating from Spain – from Murcia and from Madrid – were used, and maintained under standard conditions described in [13], [14].

*Phlebotomus perniciosus* salivary glands for immunoblots and ELISA assays were dissected from 3 to 5-day-old females, and pools of 20 salivary glands in 20 µl of Tris-NaCl buffer (20 mM Tris, 150 mM NaCl, pH 7.6) were stored at −80°C. Salivary gland homogenate (SGH) was obtained by disruption of the glands with repeated freezing and thawing cycles. Salivary glands used for isolation of mRNA were dissected from 1-day-old female sand flies, and were stored in groups of 20 salivary glands in 20 µl of RNAlater (Qiagen) and kept at −80°C until use.

### 3. Mice and canine sera

Hyperimmune mice sera were obtained by repeated exposure of three SKH1hr mice to bites of uninfected *P. perniciosus* females; three other mice of the same strain served as non-exposed controls. Canine sera from dogs (beagles) experimentally exposed to *P. perniciosus* bites were selected from those previously used for studies on SGH [7]. Sera were chosen to cover a wide range of anti-saliva antibody levels: six originated from highly exposed dogs (bitten by 150–190 sand fly females once a week), six from dogs with lower exposure (20–70 females once a week) and six from unexposed dogs (before exposure). The sera of exposed dogs were collected seven days after the fourth exposure.

### 4. Recombinant salivary proteins

Six bacterially-expressed recombinant antigens expressed in nine forms were studied: yellow protein rSP03B (KF257369), two apyrases - rSP01B (KF257364, KF257366, KF178455) and rSP01 (KF257365, KF257367), antigen 5 protein rSP07 (KF257368), ParSP25 protein rSP08 (KF178457) and D7 protein rSP04 (KF178456). Coding sequences of SP01 and SP01B (referred to further in the text as rSP01B/1 in pET42b and rSP01/1 in pET42b, respectively), SP07 and SP03B were obtained from salivary glands of *P. perniciosus* (Murcia colony). After dissection into RNAlater, mRNA was isolated by a Roche High Pure RNA Tissue Kit and transcripted to cDNA by a Roche High Fidelity cDNA Synthesis Kit using polyA primers. The second cDNA strands of SP01B and SP01 were amplified by PCR using specific primers that were synthesized according to the sequences of the mature protein (without signal peptide). These sequences have been published with the following accession numbers [15]: rSP01B/1 - DQ192491 and SP01/1 - DQ192490. Afterwards, we followed the procedure that was described in [9] – briefly, genes were expressed in pET42b (Novagen) with a His tag containing 8 histidines – the *E.coli* BL21 (DE3) expression system. In parallel, single-stranded cDNA transcripted from salivary glands as described above was sent to Apronex s.r.o. (Prague) for preparation of rSP01, rSP01B, rSP03B and rSP07 proteins in the recombinant form according to sequences published in the cDNA library [15] - rSP03B (DQ150622), rSP01B/2 (DQ192491), rSP01/2 (DQ192490), rSP07 (DQ153101). All four proteins were expressed in the *E.coli* BL21 (DE3) expression system; apyrases rSP01B/2 and rSP01/2 were expressed in the pET42b vector (Novagen) with 2 His tags - the first containing 6 histidines and the second 8 histidines, and a 1.4 kDa adaptor, while yellow protein rSP03B and antigen 5 protein rSP07 were expressed in the pET28b vector (Novagen) with 1 His tag containing 6 histidines. All proteins were isolated under denaturing conditions with 8M urea.

In addition, salivary coding sequences of SP01B, SP04 and SP08 were obtained from a cDNA library constructed from the salivary glands of *P. perniciosus* from Madrid – GenBank accession numbers are: SP01B - HE974345.1, SP04 - HE980444.1, SP08 - HE974347.1; in contrast to the other proteins tested, they contain signal sequences (thus they seem to be about 3 kDa heavier on immunoblots). Recombinant proteins rSP01B and rSP04 were expressed in the pQE31 vector (Qiagen) with a His tag containing 6 histidines in *E. coli* M15 cells, and purified under denaturing conditions with 8M urea. Protein rSP08 was cloned into the pGEX4T3 vector (Amersham Biotech), expressed in *E. coli* Arctic Express cells (Agilent), and purified by polyacrylamide gel extraction in PBS. As rSP08 was expressed as a fusion protein with gluthathione S-transferase (GST), the latter was also obtained and used as a control in immunoblots and ELISA. In all these cases, protein refolding was performed using dialysis against PBS using SnakeSkin Dialysis Tubing (10 kDa MWCO, Thermo Scientific Goettiengen, Germany).

The concentration of all proteins was quantified by the Lowry method (Bio-Rad) following the manufacturer's protocol.

### 5. Immunoblots

The immunogenicity of the recombinant proteins was tested by the immunoblot technique. Recombinant salivary proteins were separated by SDS PAGE on 12% polyacrylamide gels under non-reducing conditions using a Mini-protean apparatus (Bio-Rad). Protein concentration was optimized using preliminary experiments; the list of proteins and their quantity per well of the 5-well comb are given in Table 1. Proteins were either stained with Coomassie Blue (Invitrogen) or transferred from the gel to nitrocellulose membranes using an iBLOT dry system (Invitrogen).

**Table 1:** Concentrations of recombinant proteins used for immunoblots (µg per well) and ELISA (µg/ml) with mice and canine sera.

	Protein	Immunoblot	ELISA mouse	ELISA dog
Denatured	Apyrase rSP01B/1 pET42b	3	6	9
	Apyrase rSP01/1 pET42b	4	6	6
	Apyrase rSP01B/2 pET42b	9	6	9
	Apyrase rSP01/2 pET42b	6	3	3
	Yellow protein rSP03B pET28b	4	3	3
	Antigen 5-related protein rSP07 pET28b	9	9	9
Refolded	Apyrase rSP01B pqE31	9	35	35
	D7-related protein rSP04 pqE31	9	18	18
	ParSP25-like protein rSP08 pGEX4T3	13	3	13

Membranes were cut into strips (area corresponding to one well was cut into 5 strips), blocked for 1–2 hours with 5% milk in Tris buffer with 0.05% Tween (Tris-Tw) and then incubated for 1 hour with either mice or canine sera diluted in Tris-Tw. Mice sera were diluted 1∶200 for denatured proteins rSP01B and rSP01 in pET42b and 1∶100 in the case of other proteins; canine sera were used at a dilution of 1∶50. After washing in Tris-Tw, the strips were incubated with peroxidase conjugated anti-mouse IgG (1∶1000, AbD Serotec) or anti-dog IgG antibodies (1∶1000, Bethyl) and reacting protein bands were visualized using the substrate solution with diaminobenzidine.

### 6. ELISA

The ELISA test described elsewhere [7], [16] was modified as follows. Covalink plates (Nunc) were coated with 100 µl of either salivary gland homogenate (40 ng of protein per well, corresponds to 1/5 of a salivary gland) or recombinant salivary protein, both in 0.1 M carbonate-bicarbonate buffer (pH 9.5), overnight at 4°C. The optimal concentrations of the recombinant proteins are given in Table 1. After washing in PBS with 0.05% Tween (PBS-Tw), plates were blocked with 6% milk in PBS-Tw for 1–2 h at 37°C.

Mice and canine sera were diluted in 2% milk PBS-Tw. Dilution of mice sera 1∶1600 was optimal for rSP03B, 1∶400 for apyrases in pET42b with 1 His tag, and 1∶200 for the other proteins tested. All canine sera were used at a dilution of 1∶50. Sera were incubated for 90 min at 37°C. Following washing cycles, plates were incubated with peroxidase-conjugated anti-mouse IgG (AbD Serotec) or anti-dog IgG (Bethyl) and the color reaction was developed in the substrate solution with orthophenylendiamine. Absorbance values (OD) were recorded at 492 nm using a Multiscan RC ELISA reader (Labsystems).

### 7. Statistical analysis

The non-parametric Spearman test was used to assess correlations between total anti-SGH and anti-recombinants antibody levels in GraphPad Prism version 6 (GraphPad Software, Inc., San Diego, CA). Statistical significance was considered when the p-value was <0.05.

### 8. Accession numbers

The sequences of apyrases rSP01B/1 (in pET42b), rSP01B/2 (in pET42b), rSP01/1 (in pET42b) and rSP01/2 (in pET42b), yellow protein rSP03B (in pET28b) and antigen 5 protein rSP07 (in pET28b) were based on sequences from a published cDNA library of *P. perniciosus* [15]: rSP01B - DQ192491, rSP01 - DQ192490, rSP03B - DQ150622) and rSP07 - DQ153101. The other sequences were published directly in GenBank: rSP01B (in pQE31) - HE974345.1, rSP04 (in pQE31) - HE980444.1, rSP08 (pGEX4T3 ) - HE974347.1. The expressed sequences were published with the following GenBank accession numbers: yellow protein rSP03B (KF257369), two apyrases - rSP01B (KF257364, KF257366, KF178455) and rSP01 (KF257365, KF257367), antigen 5 protein rSP07 (KF257368), ParSP25 protein rSP08 (KF178457) and D7 protein rSP04 (KF178456).

## Results

### 1. Immunoblots with mice and canine sera

**Figure 1:**
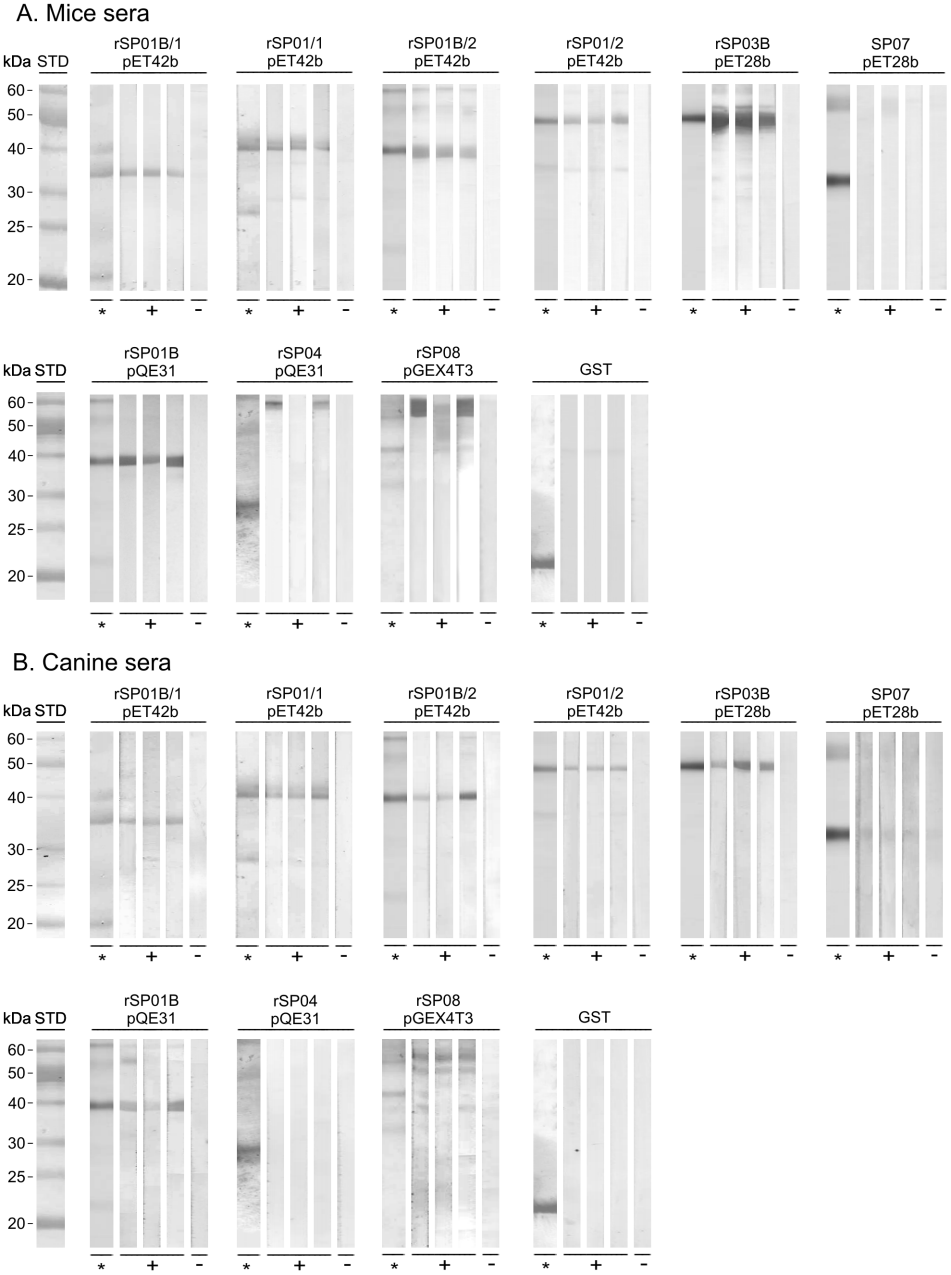
Reactivity of recombinant *P. perniciosus* salivary proteins with mice and canine sera using immunoblots. Six recombinant proteins from *P. perniciosus* saliva - yellow protein rSP03B, apyrases rSP01B and rSP01, antigen 5 protein rSP07, ParSP25 protein rSP08 and D7 protein rSP04, expressed in different vectors (pET28b, pET42b, pQE31, pGEX4T3) and a GST tag were tested. Apyrases in pET42b are expressed with either 1 His tag (rSP01/1 and rSP01B/1) or 2 His tags (rSP01/2 and rSP01B/2). Proteins were separated by SDS-PAGE and stained by Coomassie Blue (*) or incubated with mice and canine sera. (**A**) Reaction with sera from three SKH1-hr mice experimentally bitten by *P. perniciosus* females (+) and one non-exposed mouse (−). (**B**) Reaction with sera from three beagles experimentally bitten by *P. perniciosus* (+) and pre-immune serum (−).

All recombinant proteins except antigen 5 protein rSP07 and D7 protein rSP04 were recognized by the sera of all three repeatedly exposed mice; control sera and the GST tag were negative (Fig. 1A). A similar reactivity of recombinant antigens was found with the sera of the three dogs repeatedly exposed to *P. perniciosus* (Fig. 1B): all recombinant proteins except antigen 5 protein rSP07 and D7 protein rSP04 were recognized by the sera of repeatedly exposed dogs. In comparison with mice sera, the reaction of canine sera was less intense for some proteins (yellow protein rSP03B and the apyrases rSP01B/2 and rSP01/2 in pET42b) and fewer nonspecific bands appeared in the immunoblots. Control canine sera were negative (Fig. 1B).

### 2. ELISA with mice sera

**Figure 2:**
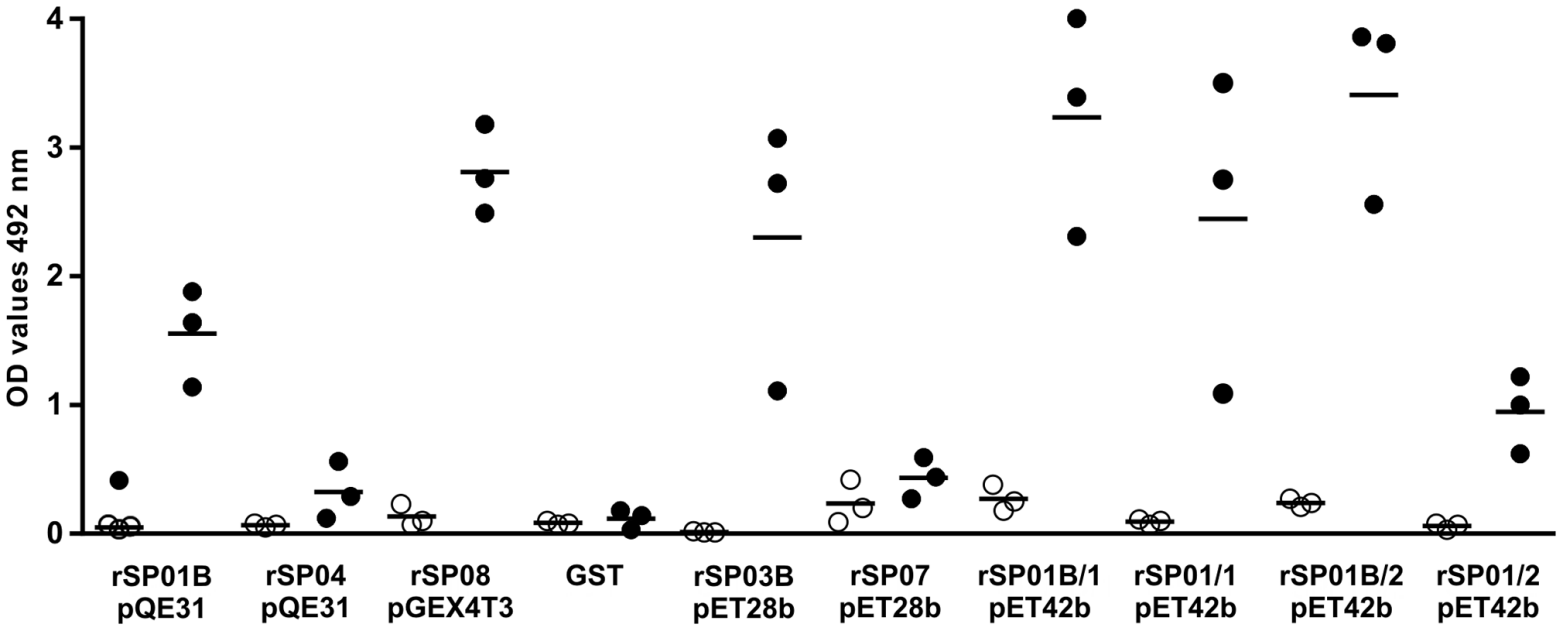
ELISA reactivity of recombinant *P. perniciosus* salivary proteins with mice sera. Six recombinant proteins from *P. perniciosus* saliva - yellow protein rSP03B, apyrases rSP01B and rSP01, antigen 5 protein rSP07, ParSP25 protein rSP08 and D7 protein rSP04, expressed in different vectors (pET28b, pET42b, pQE31, pGEX4T3) and GST tag were tested. Apyrases in pET42b are expressed with either 1 His tag (rSP01/1 and rSP01B/1) or 2 His tags (rSP01/2 and rSP01B/2) Proteins were incubated with sera with sera from three SKH1 mice experimentally bitten by *P. perniciosus* females (black circles). Non-exposed sera of three SKH1-hr mice were used as controls (white circles). Bars show means of optical density values of all three exposed and non-exposed sera. OD = optical density.

The sera of three bitten and three non-bitten mice were tested by ELISA for the presence of antibodies against the recombinant salivary proteins as well as for the anti-SGH antibodies. Results are summarized in Fig. 2. Bitten mice had a highly elevated antibody response to the following seven recombinant proteins: apyrase rSP01B in all three plasmids, both rSP01 apyrases, yellow protein rSP03B and ParSP25-like protein rSP08. Despite the low number of sera samples tested, five of these seven proteins also showed significant positive correlations with the antibody response to total SGH (rSP01B/1 in pET42b: r = 0.94, p = 0.017; rSP01B in pQE31: r = 0.94, p = 0.017; rSP01/1 in pET42b: r = 0.9, p = 0.033; rSP03B: r = 0.93, p = 0.017; rSP08: r = 1.0, p = 0.003). The sixth and seventh proteins - rSP01B/2 and rSP01/2 in pET42b - showed positive correlations but were not significant (r = 0.77, p = 0.103 for both of them).

### 3. ELISA with canine sera

**Figure 3:**
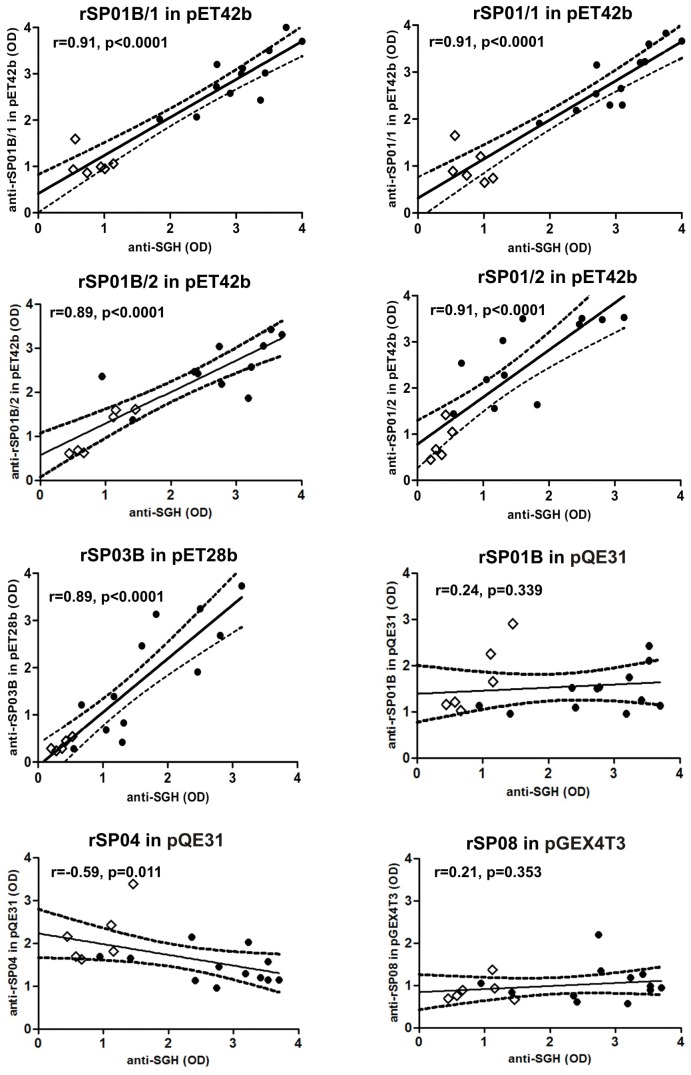
ELISA reactivity of recombinant *P. perniciosus* salivary proteins with canine sera. Six recombinant proteins from *P. perniciosus* saliva - yellow protein rSP03B, apyrases rSP01B and rSP01, antigen 5 protein rSP07, ParSP25 protein rSP08 and D7 protein rSP04, expressed in different vectors (pET28b, pET42b, pQE31, pGEX4T3) and GST tag were tested. Apyrases in pET42b are expressed with either 1 His tag (rSP01/1 and rSP01B/1) or 2 His tags (rSP01/2 and rSP01B/2). Proteins were incubated with sera from twelve beagles experimentally bitten by *P. perniciosus* females (black circles). Pre-immune sera of six beagles were used as the controls (white circles). Correlations between the levels of anti-SGH IgG and the levels of anti-recombinant proteins IgG were performed using a Spearman non-parametric test. OD = optical density, r = correlation index, p = p-value.

The ELISA results of recombinant proteins with eighteen canine sera (covering a wide range of anti-SGH antibody levels) are given in Fig. 3. Highly positive correlations with the reaction against SGH were obtained for the two denatured apyrases rSP01B and rSP01 (for both proteins in pET42b with 1 His tag: r = 0.91, p<0.0001; for rSP01B with 2 His tags r = 0.89, p<0.0001; and for rSP01 with 2 His tags r = 0.91, p<0.0001) and yellow-related protein rSP03B (r = 0.89, p<0.0001) (Fig. 3). The correlations were not significant for the other four proteins tested (Fig. 3, data not shown for antigen 5 rSP07).

## Discussion

In this study, we evaluated the reactivity of six recombinant salivary proteins of *P. perniciosus* with animal sera using immunoblots and ELISA. We chose the sera of mice as model laboratory animals and the sera of dogs as the natural reservoir host of *L. infantum*.

In immunoblots, the recombinant antigens reacted similarly with both mice and canine sera: specific reactions were achieved with both apyrases rSP01B and rSP01 (altogether 5 forms tested), yellow protein rSP03B and ParSP25 protein rSP08. On the other hand, antigen 5 protein rSP07 and D7 protein rSP04 were not recognized by any sera. The only difference between mice and canine sera was in the intensity of the positive bands – reactions with mice sera were stronger.

In ELISA, the reactivity of mice and canine sera differed in some aspects; the antibody response to refolded apyrase rSP01B, D7 protein rSP04 and ParSP25 protein rSP08 correlated with anti-SGH response only in mice sera. On the other hand, three denatured recombinant proteins, yellow protein rSP03B and two apyrases, rSP01B and rSP01, correlated significantly with the anti-SGH antibody response using both mice and canine sera. Variations in antigen conformation (denatured vs. refolded) may have led to the exposure of different epitopes. The discrepancy between results found for the refolded rSP01B and rSP08 using ELISA vs. immunoblot could be explained by differences in the exposure of antigens in these techniques; a similar lack of concordance has already been observed between ELISA and immunoblots with mice antibodies against *Phlebotomus sergenti* saliva [17].

Yellow-related proteins were found in the saliva of all sand fly species studied [18], [19], [20], [21]. They were shown to have hemagglutination and lectin-like properties [22]. They also act as high affinity binders of proinflammatory biogenic amines such as serotonin, catecholamines and histamine, suggesting that these proteins may reduce inflammation during sand fly blood-feeding [23]. In *L. longipalpis*, yellow-related protein LJM11 has been proven to have immunogenic properties leading to protective cellular immunity in C57BL/6 mice against leishmaniasis caused by *L. major* [23], [24]. Recombinant yellow-related proteins from *P. papatasi* and *L. longipalpis* reacted with the sera of hosts bitten by these sand flies [9], [10], [11]. Similarly, we have shown here that anti-*P. perniciosus* antibodies also strongly recognize recombinant yellow-related protein from *P. perniciosus* (Figures 1–3). Thus, yellow-related proteins appear to be, in general, promising markers of sand fly exposure.

Apyrases are nucleoside triphosphate-diphosphohydrolases ubiquitously present in the saliva of blood-sucking arthropods. They hydrolyze ADP and ATP in a Ca^2+^-dependent manner and inhibit ADP-induced platelet aggregation and inflammation to facilitate the blood feeding [18]. In sand fly host models, mouse and hamster antibodies elicited by *P. duboscqi* or *P. perniciosus* saliva recognized bacterially expressed apyrases of *P. duboscqi* and *P. perniciosus*, respectively [25], [26].

The three recombinant salivary proteins from *P. perniciosus* are primarily designed for measuring the canine exposure to bites of this sand fly in endemic areas of visceral leishmaniases, and for estimating the risk of *L. infantum* transmission to dogs. Seven sand fly species belonging to the subgenus *Larroussius* are proven or probable vectors of *L. infantum* in the Mediterranean area, with five of them being the most important: *P. perniciosus*, *P. ariasi*, *P. perfiliewi*, *P. neglectus* and *P. tobbi* [5], [27]. Among them, *P. perniciosus* is the most abundant in the Western Mediterranean at lower altitudes - in Italy, France, Spain and Portugal. In some of these localities, *P. perniciosus* occurs sympatrically with other *Larroussius* vectors, namely *P. ariasi*, *P. perfiliewi* and *P. neglectus* [27], [28], [29], [30], [31]. Studies on the cross-reactivity of anti-*P. perniciosus* antibodies with the saliva of these sand fly species are hampered by difficulties in the maintenance of *Larroussius* colonies; however, based on studies with other sand flies [16], [17], [32], a certain level of cross-reactivity can be expected only in closely-related species. In this case, such cross-reactivity might be an advantage as all mentioned *Larroussius* species are known to be *L. infantum* vectors [5], [27]. It is also important to point out that the specificity of the protein against sympatric sand fly species needs to be studied. Demonstrating sand fly exposure could be pivotal in the discrimination between vector-borne and direct (e.g. congenital, sexual) infections, the latter being hypothesized more and more to justify unexpected autochthonous canine leishmaniasis [33].

In conclusion, we have demonstrated that three denatured recombinant proteins from *P. perniciosus* saliva, the apyrases rSP01B and rSP01 and yellow protein rSP03B, are novel recombinant antigens with great promise in screening canine exposure to this important *L. infantum* vector and for estimating the risk of canine leishmaniases in the western Mediterranean area.
